# Dataset for bibliometric data-driven research team formation: Case of Politehnica University of Timisoara scholars for the interval 2010–2022

**DOI:** 10.1016/j.dib.2024.110275

**Published:** 2024-02-29

**Authors:** Christian-Daniel Curiac, Mihai Micea, Traian-Radu Plosca, Daniel-Ioan Curiac, Alex Doboli

**Affiliations:** a Computer and Information Technology Department, Politehnica University of Timisoara, V. Parvan 2, 300223 Timisoara, Romania; b Automation and Applied Informatics Department, Politehnica University of Timisoara, V. Parvan 2, 300223 Timisoara, Romania; c Department of Electrical and Computer Engineering, Stony Brook University, Stony Brook, NY 11794-2350, USA

**Keywords:** Bibliometrics, Team formation, Data science, Collaboration

## Abstract

Data-driven expert team formation is a complicated and multifaceted process that requires access to accurate information to identify researchers’ areas and level of expertise and their collaborative prospects. In this respect, bibliometric data represents a valuable and reliable source of information that can be effectively employed in revealing key insights regarding candidates. Due to its complex and complete structure of publication metadata records, IEEE Xplore database may offer the possibility to compute an extensive set of indicators about researchers’ publication production and how they have interacted during time. Considering the case of Politehnica University of Timisoara scholars for the interval 2010–2022, current dataset encapsulates relevant and rich information for assembling multidisciplinary research teams, being also a testing ground for experimenting and calibrating the expert team formation methods and mechanisms.

Specifications Table

**Table:** 

Subject:	Management Science, Operations Research, Library and Information Science
Specific subject area:	Expert team formation from bibliometric data
Type of data:	Sets of CSV Tables containing de-identified (using random researcher IDs) records created by consolidating raw data.
Data collection:	The raw bibliometric metadata was collected from IEEE Xplore using Metadata Search API to search for papers published by researchers from the Politehnica University of Timisoara – Romania in the time interval 2010–2022. No sampling was applied.
Data source location:	Primary data source: IEEE Xplore
Data accessibility:	Repository name: Mendeley Data Data identification number: 10.17632/r4vrvhb23h.1 Direct URL to data: https://data.mendeley.com/datasets/r4vrvhb23h/1 Instructions for accessing these data: Adhering to the appropriate citation guidelines is crucial when utilizing the dataset.

## 1 Value of the Data

• The dataset was developed to support and evaluate the use of bibliometric metadata as an authoritative and objective source of information for complex data-driven research team formation processes. It provides insightful information regarding researchers’ personal and interpersonal traits extracted from scientific production in the field of electrical engineering, computer science, and electronics. The raw data was collected for the authors affiliated with the Politehnica University of Timisoara from IEEE Xplore and corresponds to thirteen years, encompassing 1992 papers and 1179 authors.
• Besides its main purpose, the dataset may be effectively employed in evaluating research trends within a research community, revealing the evolution of the researchers or groups of researchers, or identifying effective team patterns and the mechanisms that lie behind their composition. Moreover, the dataset may be utilized to assess the research needs, track research topics that are overlooked or over-studied, and understand the scientific context to optimize institutional research-related policy- and decision-making processes.
• Some new variables are generated. In order to better evaluate individual collaborative and teamwork skills we identified the number of researcher's co-authors having the same affiliation (i.e., ‘internal collaborators’) and the number of distinct co-authors from other institutions (i.e., ‘internal ‘external collaborators’). Moreover, to reveal interpersonal affinities and their scientific results we computed four matrices, namely ‘collaborations_number’, ‘collaborations_citations’, ‘collaborations_citations_patents’, and ‘collaborations_downloads’, which respectively sum up the number of collaborations, number of citations in publications, number of citations in patents and the number of downloads the publications co-authored by pairs of researchers received.
• The dataset may be used for gaining further insights into the team selection processes by studying the effects of various factors on team outcomes, such as: a) team diversity in terms of knowledge and skills, interest, visibility level, international experience, etc.; b) researchers’ personality type; c) team dynamics and structural cohesion; d) culture sensitivity.
• The methodology and its supporting methods for compiling the data described in the current paper may be employed as a general guide when conducting similar procedures.

## 2 Background

This paper provides a dataset composed of four collections of csv tables regarding individual competencies and collaborative skills of researchers affiliated with Politehnica University of Timisoara, computed from IEEE Xplore bibliometric metadata for the period 2010–2022. Forming competent teams to fulfill multidisciplinary projects using information extracted from bibliometric databases is a relatively new approach in the quest to objectivize and optimize the research team formation processes. Starting with the pioneering work of Lappas et al. [1] which extracted information from the ‘title’, ‘authors’, and ‘year’ fields of DBLP records, little has been done to provide a deeper understanding of the insights paper metadata may offer. Since DBLP records are extremely simple [2], scholars must identify other bibliometric databases to base their data-driven team formation on. We selected IEEE Xplore to be the data source for two main reasons: the paper metadata structure is very complex, providing adequate information to assess researchers’ technical knowledge and teamwork skills [2]; and, indexing speed and open access [3]. To the best of our knowledge, the dataset made available in this paper is the first to provide valuable information extracted from a larger spectrum of metadata fields that besides the already mentioned ‘title’, ‘authors’, and ‘year’, also include ‘keywords’, ‘abstract‘, ‘affiliation’, ‘citations count’, or ‘downloads count’.

## 3 Data Description

The information provided by the four collections of data is intended to aid in identifying the researchers’ areas of expertise and assessing both their general expertise and their level of expertise within a given area. A common practice to model areas of expertise is to use their corresponding key terms [4] that may be extracted using TagMe entity linking procedure [5] from three metadata fields, namely ‘title’, ‘keywords’, and ‘abstract’. To evaluate the suitability of using such fields in devising the key terms we offer a collection of data for each of the following four cases:

• Case#1: ‘title’, ‘keywords’, and ‘abstract’ fields were used to extract 6493 key terms;
• Case#2: ‘title’ and ‘keywords’ fields were used to extract 2651 key terms;
• Case#3: ‘title’ fields were used to extract 1844 key terms;
• Case#4: ‘keywords’ fields were used to extract 1254 key terms.

Each collection consists of nine CSV tables, briefly described as follows:

**Individual expertise and collaborators** provides an overview of each anonymized author (columns of the table) in terms of the number of authored publications (row ‘publications’), number of citations (row ‘citations’), number of citations in patents (row ‘citations_patents’), number of downloads (row ‘downloads’), number of distinct co-authors having the same affiliation (row ‘internal_collaborators’), and number of distinct co-authors having other affiliations (row ‘external_collaborators’).

**Collaborations number, collaborations citations, collaborations citations patents, and collaborations downloads** provide symmetric matrices, where the rows and columns correspond to anonymized authors and the value inside a cell represents the number of co-authored publications, the number of citations received by co-authored publications, the number of citations in patents received by co-authored publications, and the number of times co-authored publications were downloaded, respectively. It is worth mentioning that the values placed on the main diagonal of these matrices correspond to the publications that the respective authors wrote.

**KeyTerms number, keyterms citations, keyterms citations patents, and keyterms downloads** provide matrices, where the rows correspond to anonymized authors, the columns correspond to identified key terms using the TagMe procedure and the value inside a cell represents the number of publications the author has produced, the number of citations received by authored publications, the number of citations in patents received by authored publications, and the number of times authored publications were downloaded, respectively.

All the values presented in the CSV tables are discrete quantitative values (whole numbers) or key terms representing Wikipedia mentions.

**Fig. 1:**
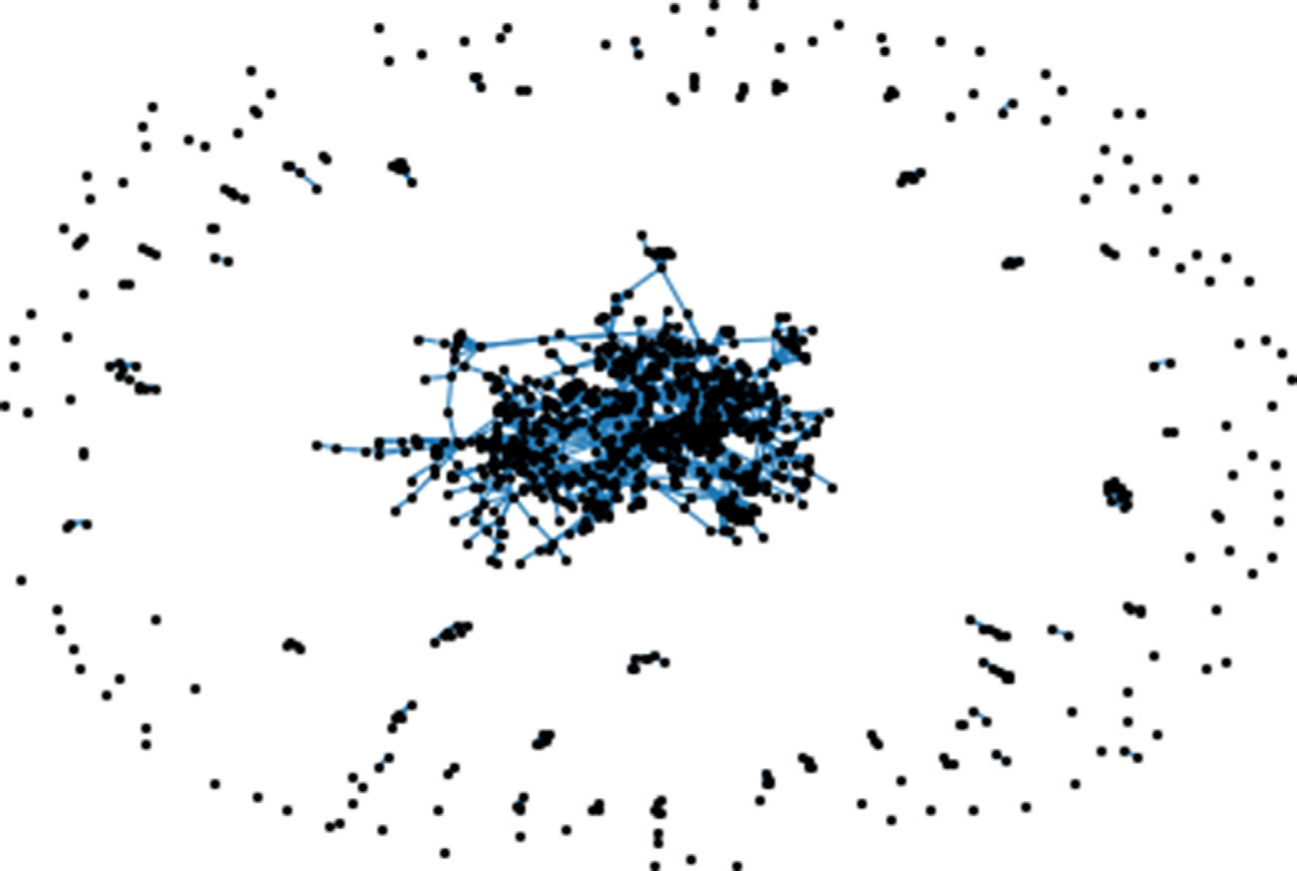
Collaborative ties between PUT scholars.

As presented in Fig. 1, the collaborative graph obtained using the information provided by Collaborations_number.csv is extremely sparse, reflecting the research work in small teams or even isolated teams. This will make the research team formation process for a given scientific theme extremely challenging due to psychological aspects including team cohesion and team members’ satisfaction.

The entire dataset is accessible from Mendeley Data at https://doi.org/10.17632/r4vrvhb23h.1 [6] and can be used in a variety of team formation problems, a typical example being the building of a multidisciplinary team to fulfill a research theme described by a finite set of key terms. In this case, the candidate's areas of expertise can be directly extracted from ‘KeyTerms_*.csv’ files, candidate's general and theme-related expertise can easily be derived from ‘Individual_Expertise_and_Collaborators.csv’ and ‘KeyTerms_*.csv’ tables, while the candidate's collaborative skills can be evaluated using the structured information about candidate's previous collaborations found in ‘Collaborations_*.csv’ files.

## 4 Experimental Design, Materials and Methods

### 4.1 Raw data collection

**Fig. 2:**
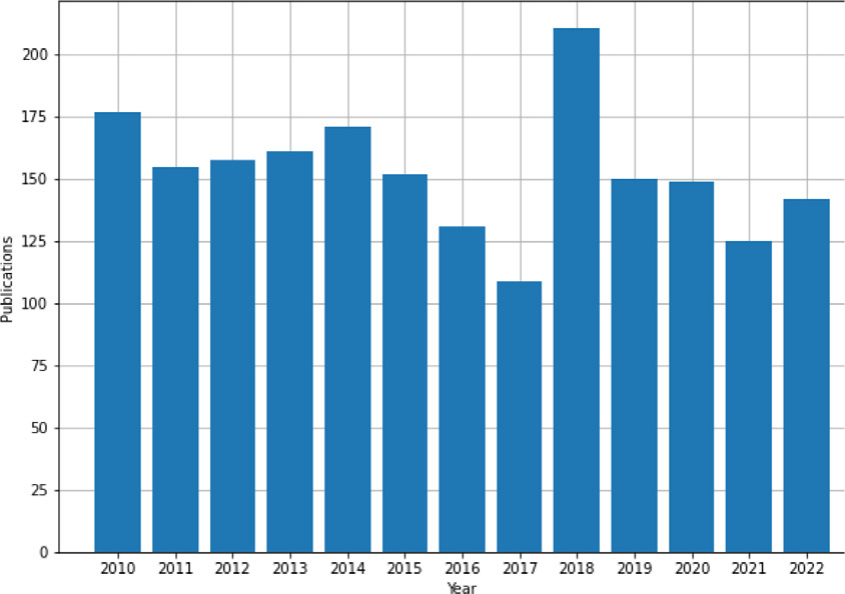
Publications per year.

**Fig. 3:**
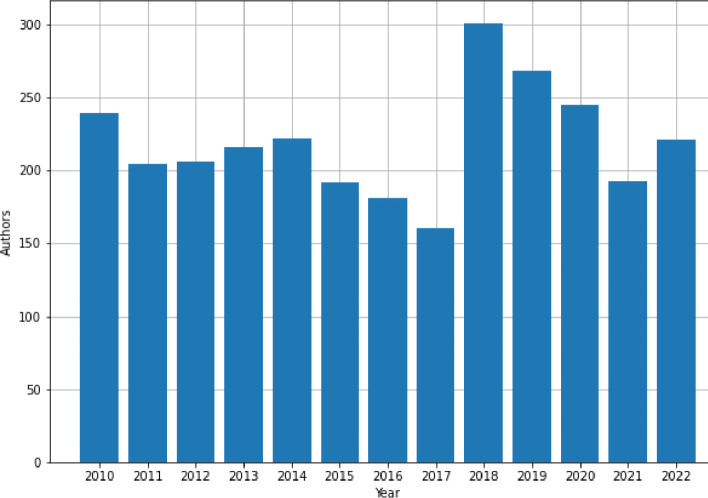
Unique authors for each year.

Using the Metadata Search API [7] to retrieve papers published by authors from the Politehnica University of Timisoara – Romania in the interval 2010–2022, 1992 bibliometric records containing ’doi’, ’title’, ’authors’, ’content type’, ’abstract’, ’publication year’, ‘citing paper count’, ’citing patent count’, ’download count’, and, ’index terms’ fields were extracted on July 4, 2023, from IEEE Xplore. This corpus covers publications authored by 1179 researchers from the mentioned university, the distribution of the number of papers and unique authors per year being presented in Figs. 2 and 3, respectively.

### 4.2 Raw data processing

In order to remove personally identifiable data in our dataset, the identities of all authors have been anonymized, using generic names of form IDx.

• To identify the key terms that define the areas of expertise, we applied the TagMe entity-linking procedure with a linking probability lp=0.1 for the concatenated field ‘title’+’abstract’+’index terms’ (Case#1), the concatenated field ‘title’+’index terms’ (Case#2), the field ‘title’ (Case#3), and field ‘index terms’ (Case#4). We selected TagMe for its effectiveness in short text processing and its flexibility in selecting the level of coarse-graining of the key terms to be considered [5].
• To display the individual expertise-related features of the authors (rows ‘publications’, ‘citations’, ‘citations_patents’, and, ‘downloads’ in Individual_Expertise_and_Collaborators.csv table), we summed up the corresponding values for all their publications.
• To identify the authors internal and external collaborators (rows ‘internal_collaborators’, and, ‘external_collaborators’ in Individual_Expertise_and_Collaborators.csv table) we extracted the affiliation (i.e., part of the ‘author’ field of IEEE Xplore paper matadata) of each of their co-authors and count the number of unique co-authors having the same affiliation and the number of distinct co-authors from other institutions.
• To form the matrix presented in the Collaborations_number.csv table we counted the number of the papers co-authored by each pair of researchers, while for the matrices presented inside Collaborations_citations.csv, Collaborations_citations_patents.csv, and Collaborations_downloads.csv tables we respectively summed up the number of citations in papers, the number of citations in patents, and, the number of downloads for their co-authored publications.
• To form the matrix presented in the KeyTerms_number.csv table we counted the number of the papers authored by each researcher where a given key term appears, while for the matrices presented inside KeyTerms_citations, KeyTerms_citations_patents, and KeyTerms_downloads csv tables we respectively summed up the number of citations in papers, the number of citations in patents, and, the number of downloads for the publications containing the key term and authored by the researcher.

It is important to mention that the key terms provided by the dataset are the ones directly extracted by TagMe method (i.e., are unfiltered).

Provided dataset will allow scholars or policymakers not only to test their team formation methods but also to identify the key information concerning researchers' expertise and their collaboration history that influence the productivity and creativity of the research teams, ultimately deriving effective, accurate, and scalable research team formation methods.

## Limitations

Not applicable.

## Ethics Statement

The required permissions have been obtained from IEEE for publishing the compiled dataset. The authors confirm that they have read and followed the ethical requirements for publication in Data in Brief and confirm that the current work does not involve human subjects, animal experiments, or any data collected from social media platforms.

## CRediT authorship contribution statement

**Christian-Daniel Curiac:** Conceptualization, Methodology, Software, Data curation, Validation, Investigation, Writing – original draft, Visualization. **Mihai Micea:** Conceptualization, Validation, Resources, Project administration. **Traian-Radu Plosca:** Software, Writing – original draft, Visualization. **Daniel-Ioan Curiac:** Methodology, Validation, Writing – original draft, Project administration, Supervision. **Alex Doboli:** Conceptualization, Methodology, Validation, Writing – review & editing, Supervision.

## Data Availability

Dataset for Bibliometric Data-Driven Research Team Formation (Original data) (Mendeley Data).
